# MicroRNA-21 (miR-21) Regulates Cellular Proliferation, Invasion, Migration, and Apoptosis by Targeting *PTEN*, *RECK* and *Bcl-2* in Lung Squamous Carcinoma, Gejiu City, China

**DOI:** 10.1371/journal.pone.0103698

**Published:** 2014-08-01

**Authors:** Long-feng Xu, Zhi-ping Wu, Yan Chen, Qi-shun Zhu, Shoeleh Hamidi, Roya Navab

**Affiliations:** 1 Key Laboratory for Animal Genetic Diversity and Evolution of High Education in Yunnan Province, Yunnan University, Kunming, Yunnan, China; 2 Tumor Institute, the 3^rd^ Affiliated Hospital of Kunming Medical University, Kunming, China; 3 Northeast Missouri Health Co, Kirksville, Missouri, United States of America; 4 University Health Network, Ontario Cancer Institute and Campbell Family Institute for Cancer Research, Toronto, Ontario, Canada; Sun Yat-sen University Medical School, China

## Abstract

**Background:**

In South China (Gejiu City, Yunnan Province), lung cancer incidence and associated mortality rate is the most prevalent and observed forms of cancer. Lung cancer in this area is called Gejiu squamous cell lung carcinoma (GSQCLC). Research has demonstrated that overexpression of miR-21 occurs in many cancers. However, the unique relationship between miR-21 and its target genes in GSQCLC has never been investigated. The molecular mechanism involved in GSQCLC must be compared to other non-small cell lung cancers in order to establish a relation and identify potential therapeutic targets.

**Methodology/Principal Findings:**

In the current study, we initially found overexpression of miR-21 occurring in non-small cell lung cancer (NSCLC) cell lines when compared to the immortalized lung epithelial cell line BEAS-2B. We also demonstrated that high expression of miR-21 could increase tumor cell proliferation, invasion, viability, and migration in GSQCLC cell line (YTMLC-90) and NSCLC cell line (NCI-H157). Additionally, our results revealed that miR-21 could suppress YTMLC-90 and NCI-H157 cell apoptosis through arresting cell-cycle at G_2_/M phase. Furthermore, we demonstrated that *PTEN*, *RECK* and *Bcl-2* are common target genes of miR-21 in NSCLC. Finally, our studies showed that down-regulation of miR-21 could lead to a significant increase in *PTEN* and *RECK* and decrease in *Bcl-2* at the mRNA and protein level in YTMLC-90 and NCI-H157 cell lines. However, we have not observed any remarkable difference in the levels of miR-21 and its targets in YTMLC-90 cells when compared with NCI-H157 cells.

**Conclusions/Significance:**

miR-21 simultaneously regulates multiple programs that enhance cell proliferation, apoptosis and tumor invasiveness by targeting *PTEN*, *RECK* and *Bcl-2* in GSQCLC. Our results demonstrated that miR-21 may play a vital role in tumorigenesis and progression of lung squamous cell carcinoma and suppression of miR-21 may be a novel approach for the treatment of lung squamous cell carcinoma.

## Introduction

Lung cancer is the most common cause of cancer-related death worldwide [1]. Despite years of research, the prognosis for patients with lung cancer remains dismal. Non-small cell lung carcinoma (NSCLC) accounts for approximately 85% of all lung cancers and less than 15% of diagnosed patients will survive longer than 5 years [2]. Gejiu City, Yunnan Province in South China is an area with high incidence and mortality rate of squamous cell lung carcinoma. Lung cancer in this area is called Gejiu squamous cell lung carcinoma (GSQCLC). The collected data displayed that the average annual incidence and mortality rate of lung cancer in male tin miners were 187.7/10^5^ and 161.0/10^5^ from 1954 to 2002 [3] in this region. In particular, the mortality rate was far above China’s standardized mortality rate (39.1/10^5^) and the world standardized mortality rate (31.2/10^5^) [4]. GSQCLC was investigated by the National Cancer Institute (NCI) and the Chinese Academy of Sciences (CAS) for its significant regional difference and profession specificity [5], [6]. Etiology and epidemiology evidence had shown that the genesis of GSQCLC was related to the complex effects of Radon (Rn), Arsenic (As), and mine dust [7], [8], [9], [10]. However, Laurer’s research considered that GSQCLC was associated with lead (Pb) content by measuring lead levels in the bones of tin miners [11]. Although numerous advances in GSQCLC therapy have been made, the survival rate of the patients is still poor. Therefore, it is urgent to develop novel and effective therapeutic strategies.

GSQCLC cell line, YTMLC-90, was established and named by Mao in 1994 [12]. This cell line was derived from a male patient who was 79 years old and had been working as a tin miner for 17 years and diagnosed with squamous cell lung carcinoma (T2N0M0) according to the TNM classification. YTMLC-90 has since been used in various research studies including antitumor drug screening and cancer molecular targeted therapy [13], [14].

MicroRNA (miRNAs) alterations are involved in the initiation and progression of human cancer [15]. miRNAs belong to a large family of endogenous small molecular RNAs which are encoded by genomes in higher eukaryotes and post-transcriptionally regulate gene expression [16]. Evidence verifies that each miRNA regulates multiple target genes, and 52.5% of miRNAs are located in cancer-associated genomic regions [17], [18], [19]. Therefore, a large number of miRNAs have already been described in the literatures as potential dagnostic and therapeutic targets for cancer. As an oncomiR, miR-21 is a potential therapeutic target in lung cancer and involved in tumor progression [20]. Additionally, overexpression of miR-21 has been found in NSCLC [21]. In our study, we revealed the expression level and therapeutic potential of miR-21 in YTMLC-90. In addition, our study showed that phosphatase and tensin homologue deleted from chromosome 10 (*PTEN*), reversion-inducing-cysteine-rich protein with kazal motifs (*RECK*) and B-cell lymphoma 2 (*Bcl-2*) are major target genes of miR-21 in NSCLC. *PTEN* is considered as a tumor suppressor gene associated with tumorigenesis and interacts with p53 pathway [22], [23]. Furthermore, *PTEN* plays a vital role in the regulation of cell cycle, inhibiting cell growth and division at the protein level [24], [25]. Recently, several reports have demonstrated that *PTEN* is involved in cell apoptosis in human hepatocellular carcinoma cells [26] and also involved in cell proliferation, migration, and invasion in gastric cancer [27]. *RECK* is thought to be a tumor invasion and metastasis suppressor gene [28], and was also found to inhibit tumor cell growth and motility in both lung and bladder cancer [29], [30]. *Bcl-2* is an oncogene and also a direct participant in the tumor cell apoptosis pathway [31]. Studies suggested that *Bcl-2* gene had been identified as a cause of some cancers, including breast, gastric and lung cancer [32], [33], [34].

In the current study, we detected the expression level of miR-21 in YTMLC-90 and NCI-H157 cells to investigate its effect on cell proliferation, invasion, migration, and apoptosis. We further explored the molecular cytogenetic characteristics and unique regulatory mechanisms between miR-21 and its target genes (*PTEN*, *RECK* and *Bcl-2*) in YTMLC-90 cells. We then investigate the precise molecular mechanism of miR-21 in YTMLC-90 and NCI-H157 cells to try to find the specificity and similarity of GSQCLC as compared to other NSCLC. Our findings revealed that miR-21 could be a novel dagnostic and therapeutic target for NSCLC. Furthermore, our study will provide a theoretical basis for lung cancer prevention, mass screening, early diagnosis and treatment in high-risk areas worldwide.

## Materials and Methods

### Plasmid Construction

The pGCMV/EGFP-hsa-miR-21 interference and the control pGCMV/EGFP-hsa-miR-NC plasmids (GenePharm, Shanghai, China) were used for transfection. The pGCMV/EGFP-hsa-miR- 21 interference plasmid: the interference fragment for pre-miR-21 was cloned into the pGCMV/EGFP plasmid between the EcoRand Age restriction sites. The control pGCMV/EGFP-hsa-miR-NC plasmid is just the plasmid alone without the interference sequence. Plasmids were purified using E.Z.N.A.™ Plasmid Maxi Kit (Omega, GA, USA). Plasmid identification was performed using sequencing and double restriction enzyme digestion (Fig. S1 in File S1).

### Cell Culture and Transfection

The human non-small cell lung cancer cell lines A549, NCI-H157, NCI-H460 and the immortalized lung epithelial cell line BEAS-2B were obtained commercially from American Type Culture Collection (ATCC). H460SM [35] was kindly provided by Dr. Ming-Sound Tsao, Princess Margaret Cancer Centre, Toronto, Ontario, Canada. The human non-small cell lung cancer cell lines YTMLC-90, XWLC-05 and GLC were detected in South China (Gejiu and Xuanwei City, Yunnan Province) and were obtained from the Tumor Institute of the 3rd Affiliated Hospital of Kunming Medical University, Yunnan, China. Cells were freshly recovered from liquid nitrogen (<6 months) and maintained in RPMI1640 (GIBCO, Grand Island, NY, USA) containing 10% fetal bovine serum (FBS, GIBCO, Cappinas, Brazil) supplemented with 100 IU/ml penicillin and 100 µg/ml streptomycin. Cells were incubated in a humidified incubator at 37°C containing 5% CO_2_.

Cells were seeded in a 6-well plate at 2×10^5^ cells/well, and then incubated in antibiotic-free Opti-MEM medium (GIBCO, Grand Island, NY, USA) for 24 hours. Cells were followed by transfection with Lipofectamine 2000 Reagent (Invitrogen, Carlsbad, CA, USA) with a final plasmid concentration of 50 ng/µl. Cells were respectively divided into 3 groups: non-transfected, pGCMV/EGFP-hsa-miR-21 interference transfected, and control negative pGCMV/EGFP-hsa-miR-NC transfected. Transfection efficiencies of approximately 65% were achieved 48 h post-transfection as assessed by Green Fluorescent Protein (GFP) using flow cytometry.

### RNA Isolation, Reverse Transcription and Quantitative Real-time PCR

Total RNA was purified using RNAprep pure Cell/Bacteria Kit (TIANGEN, Beijing, China). cDNA was generated with the iScript™ cDNA Synthesis Kit (Bio-Rad, CA, USA) combined with instructions of a specific stem-loop real-time PCR [36]. Quantitative real-time PCR was performed using iTaq™ Universal SYBR Green Supermix Kit (Bio-Rad, CA, USA) for 20 µl total reactions on the ABI 7500 System. All primers were purchased from Sangon Biotech Co., LTD (Shanghai, China). The sequences of the primers are shown in Table 1. *U6* (with a stem-loop primer) and *GAPDH* were used as reference genes. Reactions were performed in triplicate and delta-delta-Cycle Threshold (ΔΔCt) values were calculated after normalization to reference genes and converted into fold change or relative quantification (RQ) which was calculated with the 2^−ΔΔCt^ method.

**Table 1:** Primer sequences for polymerase chain reaction (PCR).

Gene name	Primer sequences (5′-3′)
Hsa-miR-21-RT^a^	GTCGTATCCAGTGCAGGGTCCGAGGTATTCGCACTGGATACGACTCAAC
Hsa-miR-21-F^b^	GCCCGCTAGCTTATCAGACTGATG
Hsa-miR-21-R^b^	GTGCAGGGTCCGAGGT
*U6*-RT^a^^,^ c	GTCGTATCCAGTGCAGGGTCCGAGGTATTCGCACTGGATACGACAAAAATATG
*U6*-F^b^^,^ c	GCGCGTCGTGAAGCGTTC
*U6*-R^b^^,^ c	GTGCAGGGTCCGAGGT
*PTEN*-F^b^	CCGAAAGGTTTTGCTACCATTCT
*PTEN*-R^b^	AAAATTATTTCCTTTCTGAGCATTCC
*RECK*-F^b^	GACTCTTCTCCTGGTCCATCTC
*RECK*-R^b^	CTATCCGTTGGGTTCCTCAT
*Bcl-2*-F^b^	ATTGTGGCCTTCTTTGAGTTCG
*Bcl-2*-R^b^	CATCCCAGCCTCCGTTATCC
*GAPDH*-F^b^^,^ c	CCAAAATCAGATGGGGCAATGCTGG
*GAPDH*-R^b^^,^ c	TGATGGCATGGACTGTGGTCATTCA

a indicates the primers are used for cDNA synthesis;

b indicates the primers are used for quantitative real-time PCR;

c indicates the primers are used as reference primers;

RT: Reverse transcription; F: Forward strand; R: Reverse strand.

### MTS Assay

Cells were transfected with 50 ng/µl of each plasmid as described above. Cells were seeded in 96-well plates at 2×10^3^ cells/well in a final volume of 200 µl. After incubation for 1, 2, 3, 4, 5, 6 and 7 days in a humidified incubator at 37°C with 5% CO_2_, 20 µl per well of MTS (3-**(**4,5-dimethylthiazol-2-yl**)**-5-**(**3-carboxymethoxyphenyl**)**-2-**(**4-sulfophenyl**)**-2H-tetrazolium, Promega, WI, USA) was added. Following 4 h incubation at 37°C, the absorbance at 490 nm was detected by Microplate Reader (Bio-Rad, Carlsbad, CA, USA).

### Wound Healing Assay

As described above, cells transfected with plasmid were trypsinized at 48 h post-transfection. Then 1×10^6^ cells were seeded into a 12-well plate in 2 ml RPMI1640 (GIBCO, Grand Island, NY, USA) with 10% fetal bovine serum (FBS, GIBCO, Cappinas, Brazil) and incubated for 24 h. Wounds were produced in the monolayer using a 20 µl tip (at time 0) [19], [37]. Then cells were washed with PBS (pH = 7.2, GIBCO, Grand Island, NY, USA) and incubated for 24 h, 36 h and 72 h. The distance between the two sides of the wound was measured with an inverted optical microscope (Olympus, Tokyo, Japan).

### *In vitro* Trans-well Invasion Assays

Cells were incubated in Opti-MEM (GIBCO, NY, USA) medium for 12 h at 48 h post-transfection. Matrigel (dilute with 7 times RPMI1640 medium) was added into the upper chambers in a volume of 60 µl. Cells were seeded in the upper chamber of the 24-well plate at a ratio of 1×10^5^ cells/well in a final volume of 200 µl using Opti-MEM medium. Then 600 µl RPMI1640 with 10% fetal bovine serum was added in the lower chamber of the trans-well plate. After incubation at 37°C in 5% CO_2_ atmosphere for 24 h, cells were fixed with 90% ethanol for 30 min and stained with 0.1% crystal violet for 1 h. The number of invasive cells was counted under the microscope (Olympus, Tokyo, Japan).

### Western Blot Analysis

Cells were transfected with 50 ng/µl of each plasmid. 48 hours later, cells of each group were lysed with RIPA lysis buffer, washed with PBS (pH = 7.2, GIBCO, Grand Island, NY, USA), and then the total proteins were extracted. The protein concentration of cell lysates was determined by BCA Protein Assay Kit (TIANGEN, Beijing, China). 30 µg sample extract was resolved on 5–10% SDS-polyacrylamide gels (separating gel, 10%, 120 V; stacking gel, 5%, 80 V) using a gel electrophoresis apparatus (Bio-Rad, CA, USA) and transferred to polyvinylidene fluoride membrane (PVDF, Millipore, MA, USA). Membranes were blocked for 1 h with 5% nonfat dry milk in Tris-buffered saline containing 0.05% Tween (TBST). Membranes were incubated overnight at 4°C with primary antibody (anti-*PTEN*, anti-*RECK*, anti-*Bcl-2* and anti-*β-actin*) (Cell Signaling, BOS, USA), and then washed and incubated with secondary antibody, horse radish peroxidase (HRP) labeled goat anti-rabbit or anti-mouse immunoglobulin G (IgG) (Santa Cruz, CA, USA). All antibodies were dissolved in Tris-buffered saline with Tween (TBST) in a ratio of 1∶1000, and finally protein expression was visualized by Enhanced Chemiluminescence (ECL). Syngene GeneGenius image acquisition (Syngene, MD, USA) and GeneTools analysis software were used to quantify the band intensities.

### Apoptosis Assay

DUTP-FITC/propidium iodide (PI) staining was performed for the detection of apoptotic cells. 48 h after transfection, 1×10^6^ cells were collected and washed twice with ice-cold PBS (pH = 7.2, GIBCO, Grand Island, NY, USA). The cells were stained using the 0.002% Triton X-100, 100 U/ml RNase and 50 µg/ml PI for 30 min at 37°C, and then were detected by flow cytometry according to the guidelines. The non-transfected cells served as a negative control. The analysis of cell cycles and cell apoptosis was measured by flow cytometry (Beckman-Coulter, CA, USA), and DNA content was analyzed by COULT WINCYCLE software. The cell cycle distribution and cell percentage at G_0_/G_1,_ G_2_/M and S phase were determined by Wincycle DNA software. More than 1×10^6^ cells were fixed with 3.5% glutaraldehyde following with 1% osmic acid. Then cells were dehydrated by increasing alcohol and acetone concentration gradually and embedded with embedding medium. The semithin section of cell samples was measured using ultra-microtome and staining with the citrate-uranium acetate. Finally, images were collected under the JEM-1011 transmission electron microscopy (JEOL, Tokyo, Japan).

### Statistical Analysis

All numerical data are presented as means ± SD and were analyzed by two-way ANOVA and Student t-tests. All reported p-values are two-sided, and a p value of less than 0.05 was considered to indicate statistical significance. All statistical tests were performed using SPSS 19.0 software.

## Results

### MiR-21 Overexpression was Found in Human NSCLC Cell Lines

**Figure 1:**
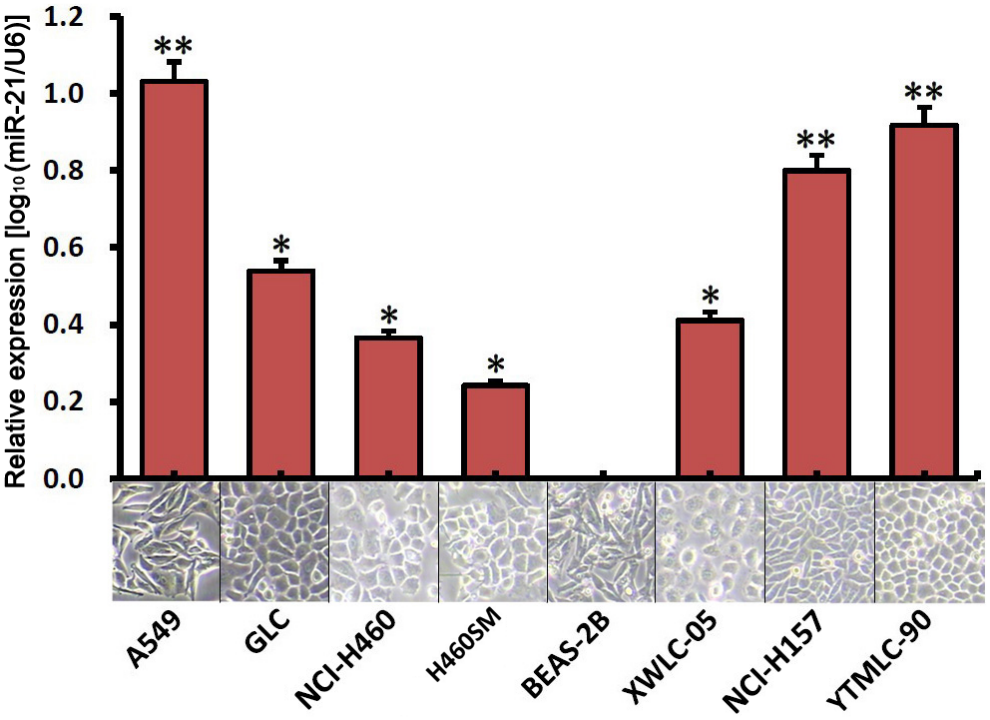
The expression level of miR-21 in the immortalized normal lung epithelial cell line BEAS-2B and non-small cell lung cancer cell lines. The expression level of miR-21 was detected by quantitative real-time PCR. miRNA abundance was normalized to *U6* as a reference gene, the expression values are normalized to BEAS-2B (n = 6, *p<0.05, **p<0.01 versus BEAS-2B). Cells images are under 100× microscopic fields.

To understand whether the expression of miR-21 was correlated with the malignancy of GSQCLC, we first evaluated the expression level of miR-21 in human NSCLC cell lines A549, NCI-H157, NCI-H460, H460SM, YTMLC-90, XWLC-05 and GLC, compared to an immortalized lung epithelial cell line BEAS-2B [38]. The result in Fig. 1 revealed that the expression level of miR-21 increased significantly in all human NSCLC cell lines (p<0.05 or p<0.01, Table S1 in File S1, the value of 1.0 means a 10-fold increase compared to BEAS-2B).

### MiR-21 Regulated the Expression Levels of *PTEN*, *RECK* and *Bcl-2* in NSCLC Cell Lines

**Figure 2:**
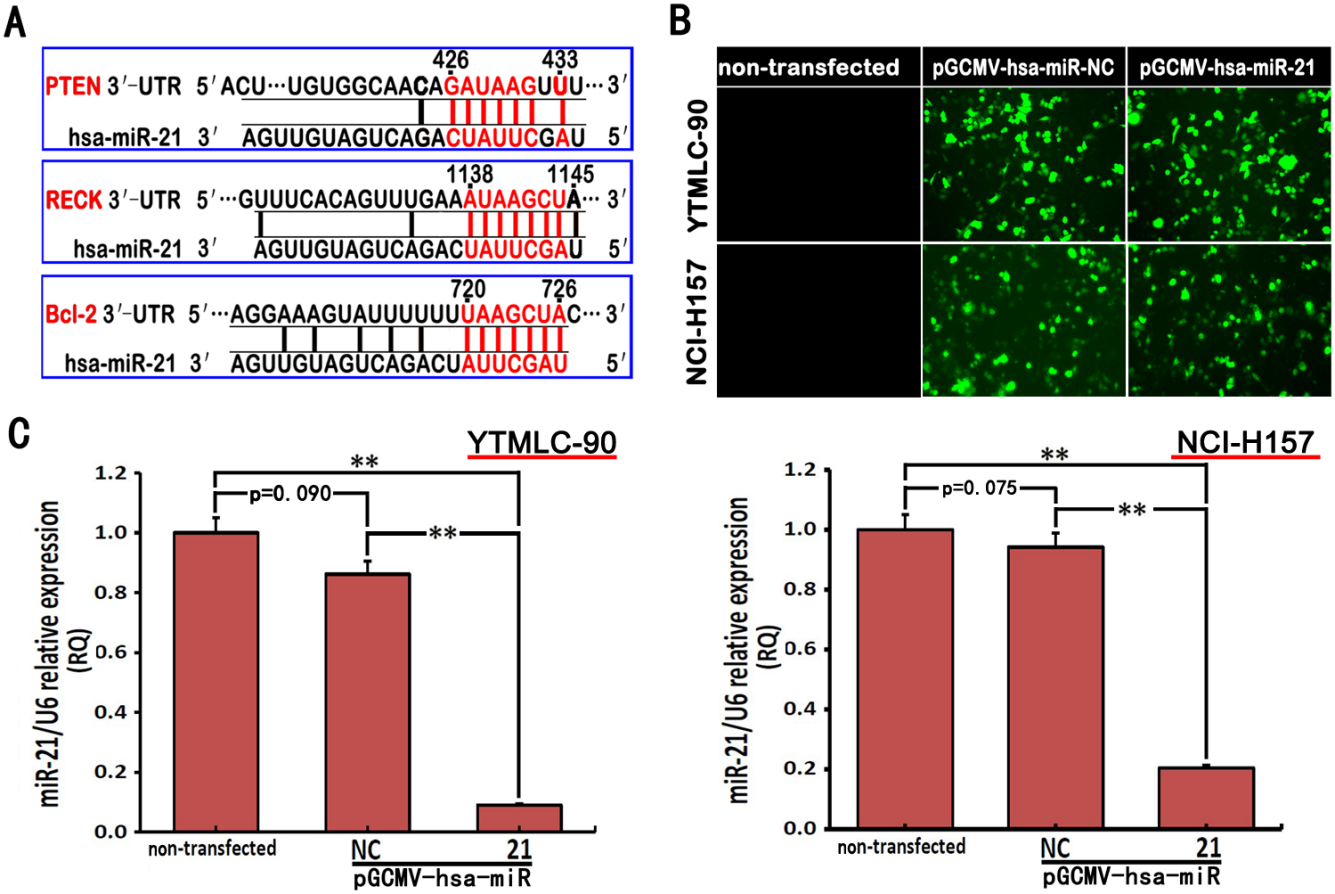
miR-21 targets gene and cell transfection efficiency were determined. **A.** The 3′-UTR of *PTEN*-mRNA, *RECK*-mRNA and *Bcl-2*-mRNA are targets for miR-21 and the seed matching sequences were marked in red. The miRNA targets were predicted by Target Scan Human Release 6.2 (http://www.targetscan.org). **B.** The expression of green fluorescent protein (GFP) transfected with pGCMV/EGFP-hsa-miR-21 or pGCMV/EGFP-hsa-miR-NC for 48 h was observed using a fluorescent inverted microscope (40×). **C.** The expression level of miR-21 was analyzed by quantitative real-time PCR at 48 h post-transfection shown in the bar graphs. miRNA abundance was normalized to *U6* as a reference gene (n = 3, **p<0.01 versus corresponding control). NC means negative control (transfected with pGCMV/EGFP-hsa-miR-NC plasmid), RQ means relative quantitation.

We selected *PTEN*, *RECK* and *Bcl-2* as potential target genes of miR-21 to study the role of miR-21 in GSQCLC (Fig. 2A). We first used pGCMV/EGFP-hsa-miR-21 interference plasmid to silence miR-21 expression. pGCMV/EGFP-hsa-miR-NC was used as a control. To evaluate the efficacy of miR-21 silencing in YTMLC-90 and NCI-H157 cells, we detected the expression level of miR-21 by real-time PCR. In addition, transfection efficiency varied based on the concentration of plasmids and was monitored by fluorescence quantification of green fluorescent protein (GFP) for both cell lines. Accordingly, we showed that pGCMV/EGFP-hsa-miR-21 interference plasmid or pGCMV/EGFP-hsa-miR-NC plasmid demonstrated a higher GFP gene transfection efficiency for both cell lines at 48 h after transfection (Fig. 2B). Furthermore, Fig. 2C and Fig. S2 in File S1 showed miR-21 expression level was significantly reduced by pGCMV/EGFP-hsa-miR-21 interference plasmid (p<0.01). Therefore, pGCMV/EGFP-hsa-miR-21 interference plasmid was selected for all subsequent studies.

**Figure 3:**
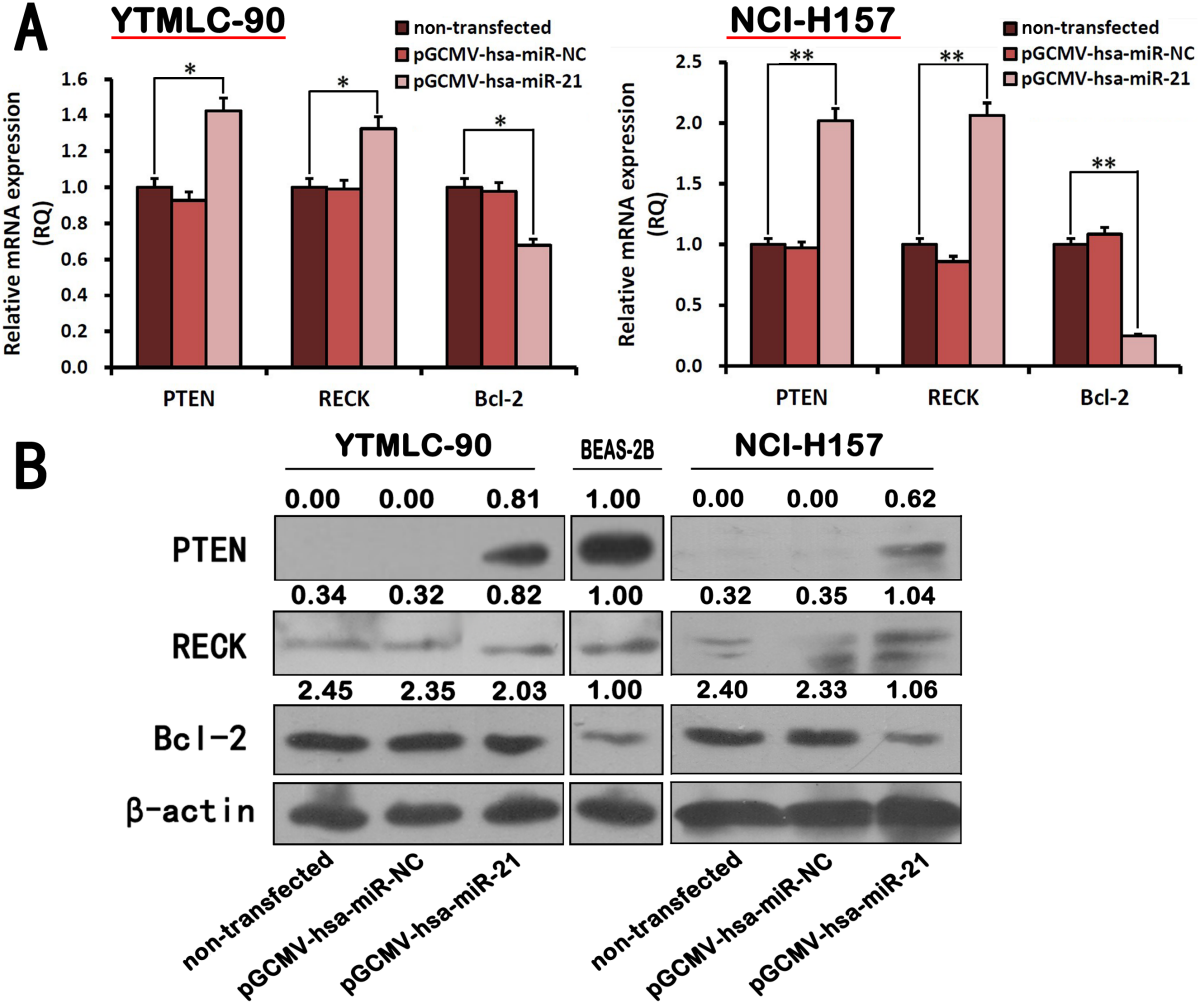
miR-21 regulated *PTEN*, *RECK* and *Bcl-2* expression in NSCLC cells. YTMLC-90 and NCI-H157 cells were transfected with pGCMV/EGFP-hsa-miR-21 or pGCMV/EGFP-hsa-miR-NC for 48 h. **A.** The mRNA expression levels of *PTEN*, *RECK* and *Bcl-2* were detected by quantitative real-time PCR in 48 h transfected cells. miRNA abundance was normalized to *GADPH* (n = 3, *p<0.05, **p<0.01 versus corresponding control). **B.** *PTEN*, *RECK* and *Bcl-2* protein level were determined after 48 h by western blot assay. The *β-actin* level was also measured as a reference gene and BEAS-2B was served as a control, NC means negative control (transfected with pGCMV/EGFP-hsa-miR-NC plasmid), RQ means relative quantitation.

In order to determine whether high expression level of miR-21 is associated with expression levels of *PTEN*, *RECK* and *Bcl-2* in YTMLC-90 and NCI-H157 cells, we performed quantitative real-time PCR and western blot analysis. The results showed miR-21 down-regulation by pGCMV/EGFP-hsa-miR-21 interference plasmid at 48 h post-transfection significantly resulted in the increase of *PTEN* and *RECK* gene expression, and the reduction of *Bcl-2* gene expression in YTMLC-90 and NCI-H157 cells at mRNA level (p<0.05, p<0.01) (Fig. 3A) or protein level (p<0.05, p<0.01) (Fig. 3B, Fig. S3 in File S1). We also found that *PTEN* protein was not expressed in YTMLC-90 and NCI-H157 cell lines, but *RECK* and *Bcl-2* (Fig. S4 in File S1). Interestingly, there was no significant difference in *PTEN*, *RECK* and *Bcl-2* gene expression in miR-21 silenced YTMLC-90 cells when compared to miR-21 silenced NCI-H157 cells. All together, these results demonstrated miR-21 could suppress *PTEN*, *RECK* but increase *Bcl-2* expression in both YTMLC-90 and NCI-H157 cells.

### MiR-21 Promoted Cell Proliferation and Viability in NSCLC

**Figure 4:**
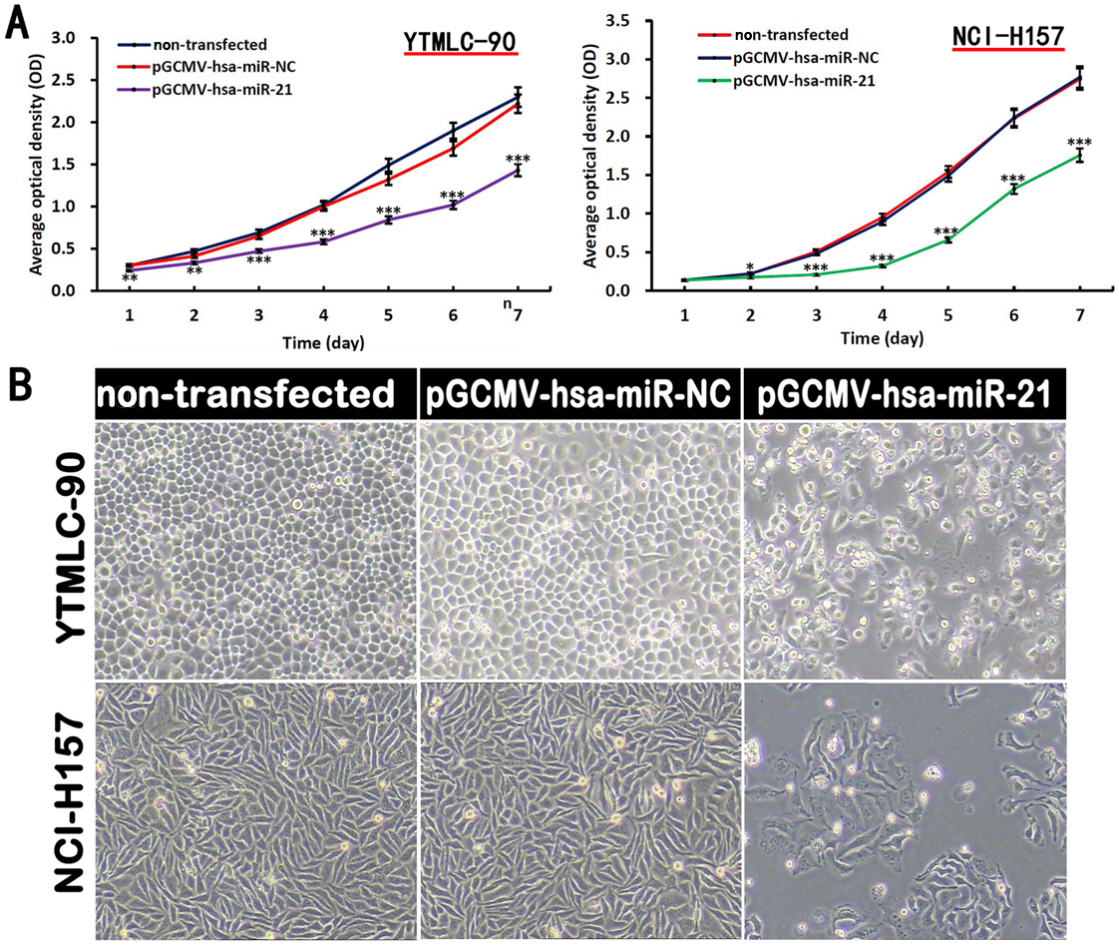
Down-regulation of miR-21 inhibited cell proliferation and viability. **A.** Cell proliferation ability was detected by MTS assay. The proliferation rate of cells transfected with pGCMV/EGFP-hsa-miR-21 interference plasmid was inhibited compared with cells in the other groups (n = 6, **p<0.01, ***p<0.001 versus corresponding control). **B.** Morphologic changes of YTMLC-90 and NCI-H157 cells was observed in response to miR-21 inhibition. Cells images are under 100× microscopic fields. NC means negative control (transfected with pGCMV/EGFP-hsa-miR-NC plasmid), RQ means relative quantitation.

In order to confirm the specific role of miR-21 on YTMLC-90 and NCI-H157 cell proliferation and viability, cells were transfected with plasmid harboring interference fragment for miR-21 (pGCMV/EGFP-hsa-miR-21) or control plasmid (pGCMV/EGFP-hsa-miR-NC). Transfected and non-transfected cells were incubated for different days and the cell proliferation was assessed by MTS assay. As shown in Fig. 4A, proliferation of cells transfected with pGCMV/EGFP-has-miR-21 interference plasmid was significantly inhibited relative to the remaining groups (p<0.01, p<0.001), and we also found the inhibitory effect on cell proliferation in NCI-H157 cells was stronger than that in YTMLC-90 cells. In addition, the images of cells demonstrated that transfected cells displayed significant reduction in adherent cell number at 24 h post-transfection. The result was concordant with the MTS data (Table S2 in File S1). miR-21 silenced cells exhibited a morphological change with cell detachment, floating, and rounding, and the rest of adherent cells showed conspicuous shrinkage, serration (for NCI-H157) and spindling (for YTMLC-90) (Fig. 4B). Altogether, these results showed silencing miR-21 could reduce NSCLC proliferation and viability.

### MiR-21 Knockdown Decreased the Capacity of both YTMLC-90 and NCI-H157 Cells for Migration and Invasion

**Figure 5:**
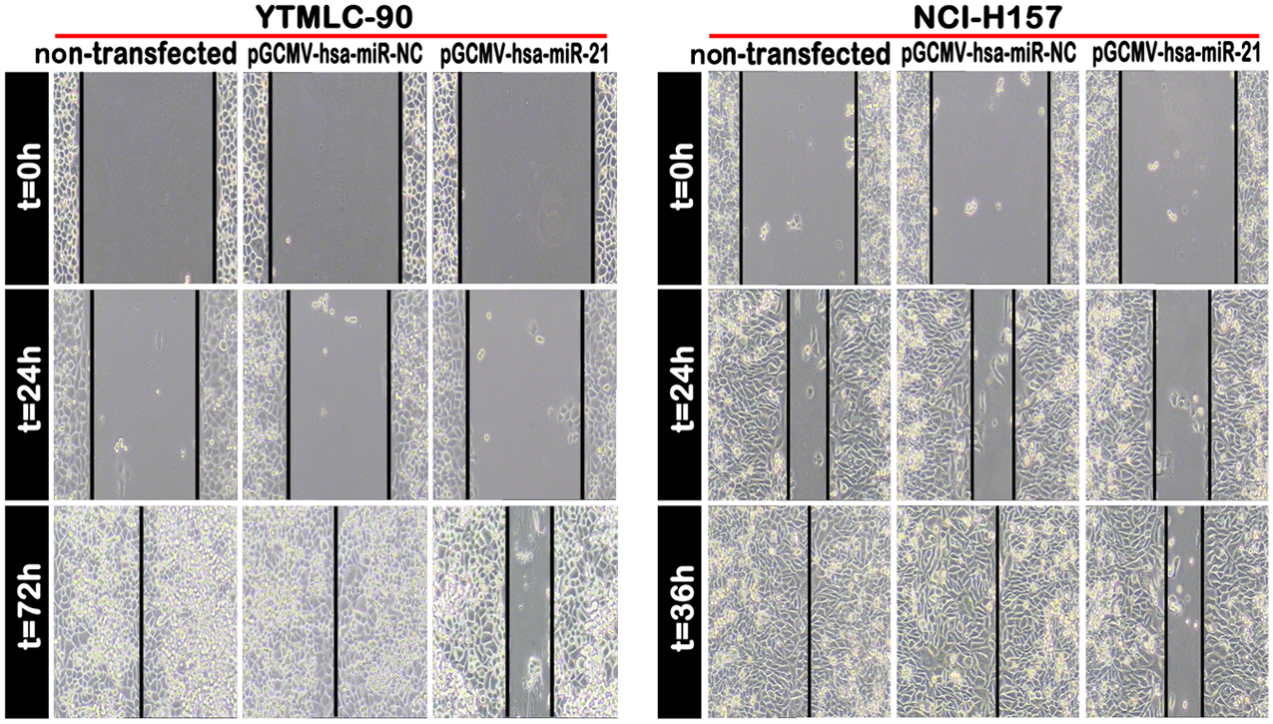
Inhibition of miR-21 expression weakened the cell migration ability (100×). Cell migration was detected using the wound healing assays. Uniform scratches were made in YTMLC-90 and NCI-H157 cells and the serial photographs were obtained at regular time of post-transfection. NC means negative control (transfected with pGCMV/EGFP-hsa-miR-NC plasmid).

**Figure 6:**
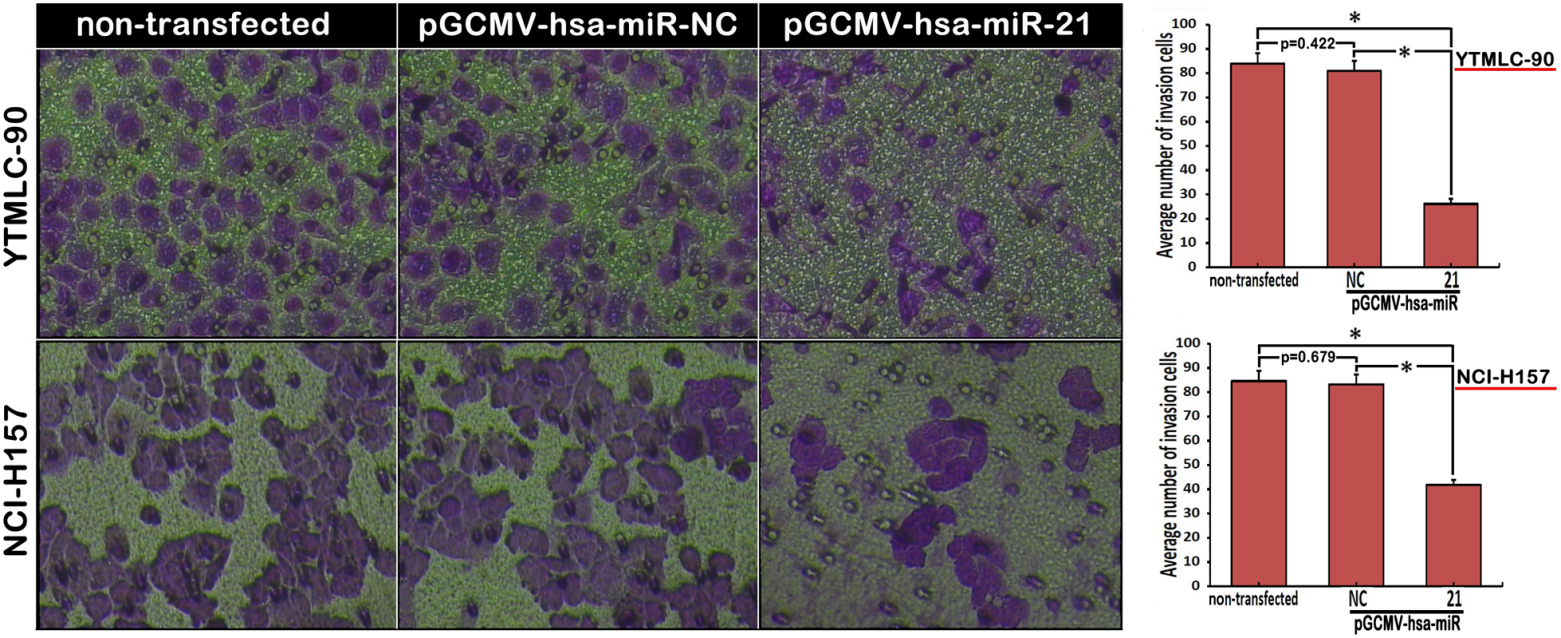
Knockdown of miR-21 suppressed cell invasion (200×). The number of invasive cells was determined by trans-well invasion assays and enumerated under the inverted microscope. The average number of invasion cells was calculated from 5 random views. Invasion cells were stained with 0.1% crystal violet and appeared in purple. NC means negative control (transfected with pGCMV/EGFP-hsa-miR-NC plasmid) and data represent means of quintuplicates ± SD (n = 5, *p<0.05 versus corresponding control).

To evaluate migratory potential of miR-21 silenced YTMLC-90 and NCI-H157 cells, wound healing assay was performed in vitro. The results revealed that tumor cells with miR-21 knockdown rapidly closed the scratch wounds compared with the control cells (p<0.001) (Fig. 5). Moreover, the wounds of the miR-21 silenced YTMLC-90 cells were still open at 36 h at this time while miR-21 silenced NCI-H157 cells had almost closed the wounds at this time, which suggested that NCI-H157 cells with miR-21 knockdown have higher migratory potential relative to YTMLC-90 cells with miR-21 knockdown. We further performed *in vitro* trans-well invasion assay to investigate the effect of miR-21 on the invasive ability of YTMLC-90 and NCI-H157 cells. As shown in Fig. 6, miR-21 knockdown resulted in increased YTMLC-90 and NCI-H157 cell invasion rate compared with the control cells (p<0.05) (Table S3 in File S1). These results clearly suggested that silencing miR-21 could inhibit migration and invasion of YTMLC-90 and NCI-H157 cells.

### Down-regulation of miR-21 Induced Cell Apoptosis *in vitro*

**Figure 7:**
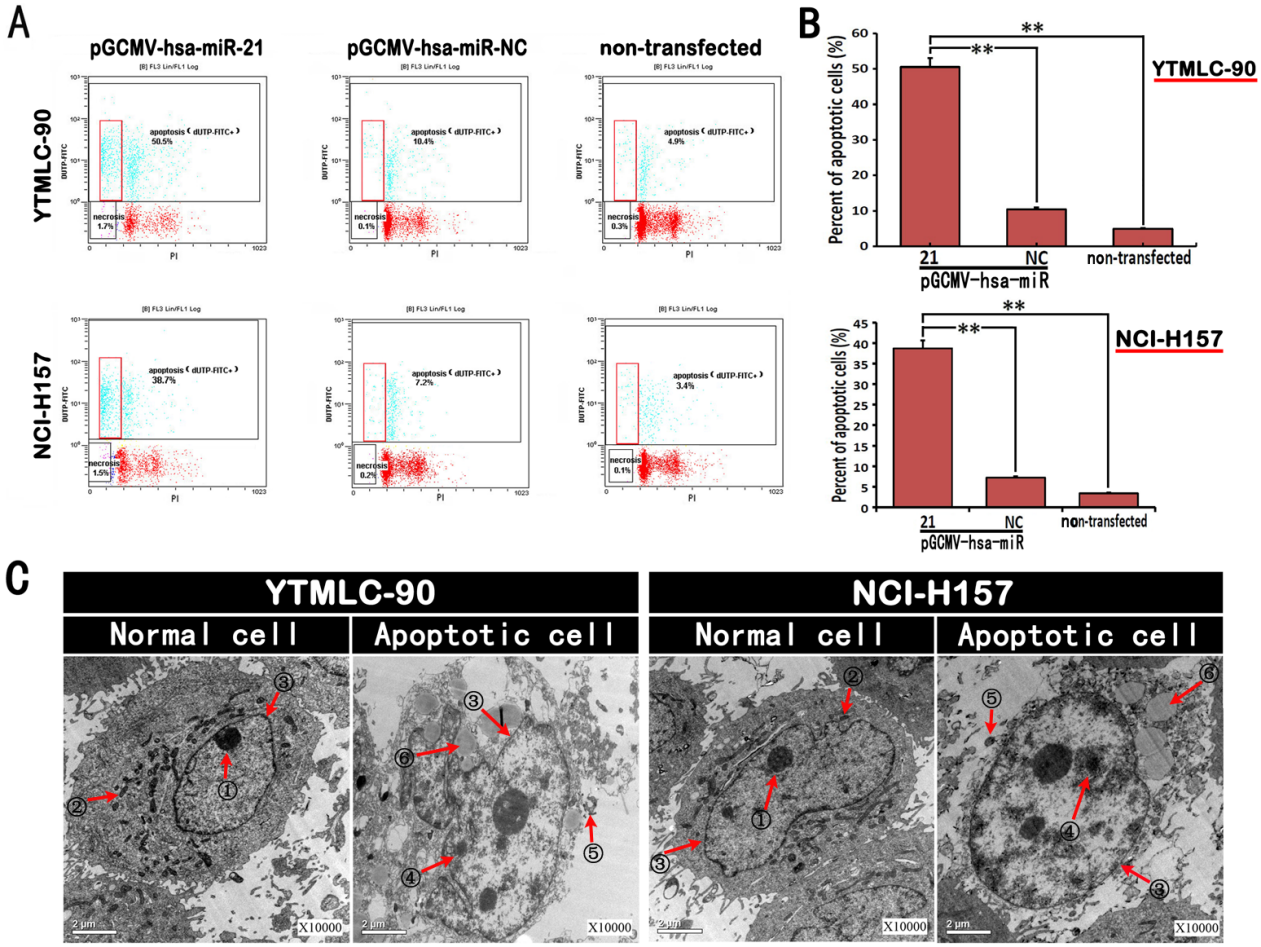
Down-regulation of miR-21 induced NSCLC cell apoptosis. **A.** Flow cytometry (FCM) method was used to analyze the apoptosis rate of cells in each group at a qualitative level. The early apoptotic cells were marked by red boxes. **B.** The percent of the apoptotic cells in each group. Data represent means of triplicates ± SD (n = 3, **p<0.01 versus corresponding control). NC means negative control (transfected with pGCMV/EGFP-hsa-miR-NC plasmid). **C.** The cell morphology of apoptotic cells relative to the normal cell morphology on a quantitative level (10000×). Normal cells were from the un-transfected groups, and the apoptotic cells were from the pGCMV/EGFP-hsa-miR-21 interference plasmid transfected groups at 48 h post-transfection. These images were collected by the transmission electron microscope (TEM), 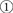
nucleolus, 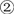
mitochondria, 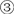
karyotheca, 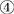
high density chromatin, 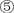
apoptotic body, 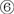
cell vacuolation.

In order to assess whether silencing miR-21 possesses proapoptotic properties, we transfected YTMLC-90 and NCI-H157 cells with pGCMV/EGFP-hsa-miR-21 interference or a negative control pGCMV/EGFP-hsa-miR-NC plasmid. Features of the apoptotic cell death were then measured by flow cytometry at 48 h post-transfection. As shown in Fig. 7A, silencing miR-21 caused a significant decrease in the number of apoptotic cells compared to the control cells. Furthermore, silencing miR-21 led to an increased rate of apoptosis in YTMLC-90 when compared with NCI-H157. The results demonstrated that the rates of apoptosis in miR-21 silenced YTMLC-90 cells and NCI-H157 were 50.5±1.2% (p<0.05) and 38.7±1.6% respectively, significantly higher than negative control (p<0.05). The percent of apoptosis in YTMLC-90 cells transfected with pGCMV-has-miR-NC was 10.4±0.6% and 7.2±0.4% for NCI-H157 cells. As another control, the percent of apoptosis in non-transfected YTMLC-90 and NCI-H157 cells was compared and found to be 4.9±0.6% and 3.4±0.3% respectively (Fig. 7B). Next, transmission electron microscopy (TEM) investigated in detail the miR-21 knockdown-induced apoptotic cell death. The ultrastructural analysis has shown that some morphological changes occurred when miR-21 was knocked down in YTMLC-90 and NCI-H157 cells. The presence of apoptotic bodies were detected in miR-21 silenced cells, confirming the occurrence of apoptotic processes (Fig. 7C). Our data demonstrated that inhibition of miR-21 promoted YTMLC-90 and NCI-H157 cell apoptosis.

### Inhibition of MiR-21 Induced Cell Cycle Arrest at G_2_/M Phase in both YTMLC-90 and NCI-H157 Cell Lines

**Figure 8:**
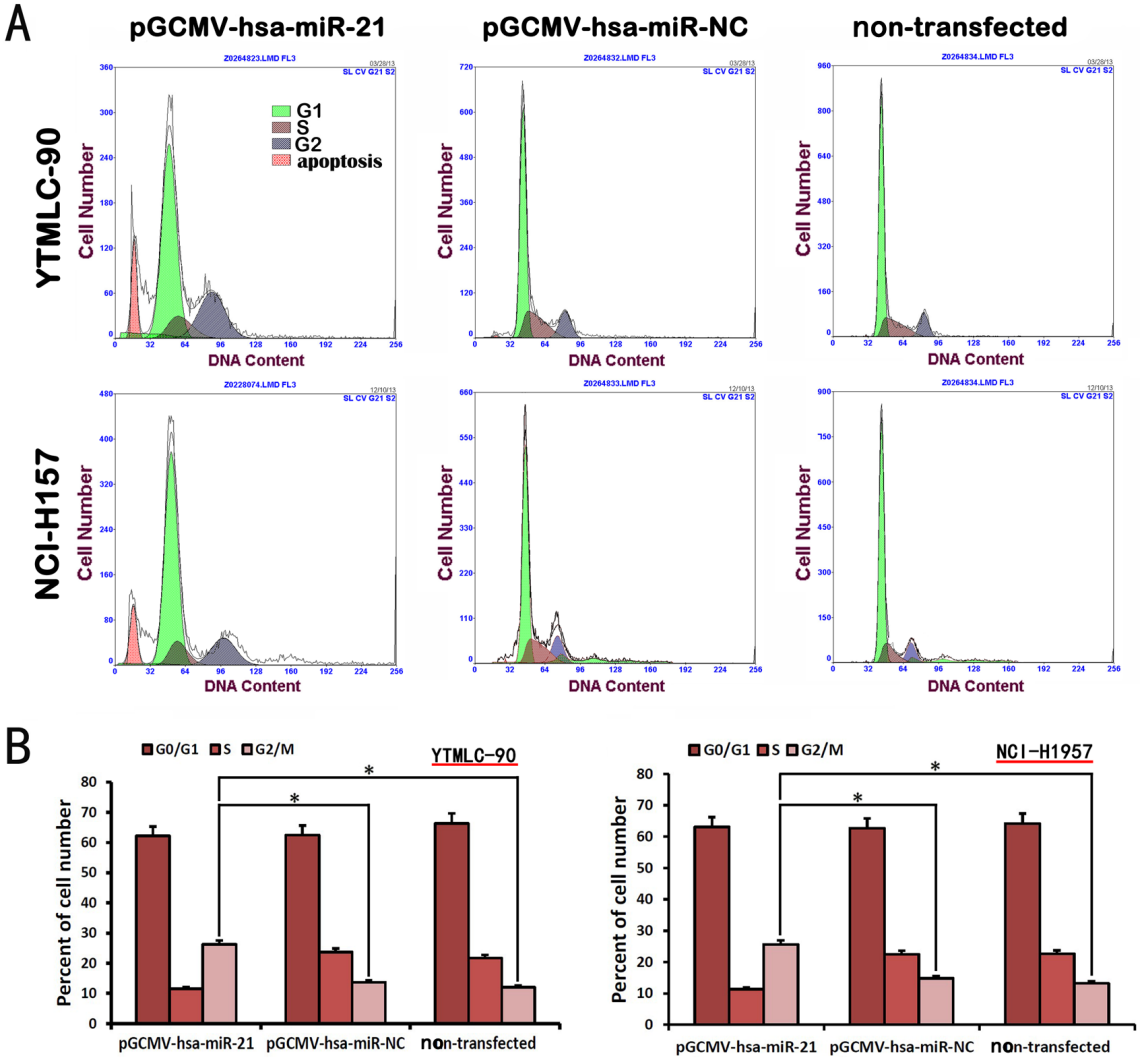
miR-21 inhibition induced cell-cycle arrest at G_2_/M phase. **A.** Cell number in each phase of cell cycle and apoptosis was detected by flow cytometry method. **B.** The percent of cell number at different phases of cell cycle (G_0_/G_1_, S and G_2_/M). NC means negative control (transfected with pGCMV/EGFP-hsa-miR-NC plasmid) and data represent means of triplicates ± SD (n  = 3, *p<0.05 versus corresponding control).

Flow cytometric analysis of the cell cycle and DNA content was performed to determine the ability of miR-21 knockdown to induce cell cycle arrest in both YTMLC-90 and NCI-H157 cell lines. Our results revealed that the sub-diploid apoptosis peak occurred in miR-21 silenced cells (Fig. 8A). Moreover, G_2_/M phase showed a significant increase in miR-21 silenced cells when compared to non-transfected cells or negative control cells (p<0.05). These results indicated that inhibition of miR-21 induced cell cycle arrest at G_2_/M phase in both YTMLC-90 and NCI-H157 cell lines.

## Discussion

Lung cancer is the leading cause of cancer death worldwide [39], [40] and a high incidence rate occurs in China, so we focus on studying lung squamous carcinoma in South China (Gejiu City, Yunnan Province; GSQCLC) for its regional difference and professional specificity. This disease has a high incidence in tin miners and attracts the attention of domestic and global scholars worldwide [41]. Numerous efforts were done to reduce the incidence and mortality of GSQCLC [7], [8], [9], [10], [11], however there is currently no effective treatments for this regional-specific disease and the precise molecular mechanism involved remains unclear. Although some therapies including tumor removal surgery, radiotherapy, and chemotherapy have been developed, the survival rate has still not improved. Therefore, developing novel strategies for GSQCLC treatment is particularly important.

Researchers have developed some anti-cancer drugs for this type of squamous cell lung carcinoma (GSQCLC). Zhu and coworkers showed that phenylbutyrate (PB) could inhibit the proliferation of squamous cell lung carcinoma cells (YTMLC-90) and induce tumor cell apoptosis [42]. Chen et al. demonstrated that brain derived neuro-trophic factor (*BDNF*) enhanced YTMLC-90 cell growth [43]. Our study for the first time investigated the regulatory mechanism between miR-21 and its target genes, including *PTEN*, *RECK* and *Bcl-2* in YTMLC-90 cells. Our goal is to identify novel potential therapeutic targets for this regional-specific squamous cell lung carcinoma based on the cellular and molecular level.

It is well known that the abnormal expression of microRNAs is related to carcinogenesis. Numerous studies observed that overexpression of miR-155 [44], miR-196a [45], miR-31 [46], as well as miR-21 play an important role in progression of lung cancer [47], [48]. Moreover, the regulation of multiple target genes by miR-21 has been experimentally validated. *PTEN* and *RECK* are important regulators of multistep tumorigenesis in lung cancer and newly identified as direct targets of miR-21. Studies demonstrated that miR-21 down-regulates the expression level of tumor suppressor gene *PTEN,* which stimulates cell growth, invasion, and metastasis in NSCLC [49], [50]. It has been shown that miR-21 modulates cell growth, invasion, and apoptosis by targeting *RECK* in many cancers, such as oral cancer [51], esophageal cancer [52], prostate cancer [53], and glioma [54], however the relationship between miR-21 and *RECK* in lung cancer is largely unknown. In our study, we evaluated whether miR-21 participates in cell growth, proliferation, invasion, metastasis, and/or apoptosis via regulating *PTEN* and/or *RECK* in the regionally-specific lung cancer GSQCLC. As an oncogene, *Bcl-2* is also a direct target of miR-21 and plays an important role in the tumor cell apopposis pathway. Previous data have shown that miR-21 targets *Bcl-2* and participates in tumorigenesis of human glioblastoma [55], bladder cancer [56], and breast cancer [57]. Our goal is to explore unclear molecular mechanisms between miR-21 and its targets (*PTEN*, *RECK, Bcl-2)* in NSCLC cells and try to reveal the specificity and similarity of GSQCLC as compared to other NSCLC.

In the current study, we observed the overexpression of miR-21 in NSCLC cell lines relative to lung epithelial cell line BEAS-2B and high expression level of miR-21 is associated with expression levels of *PTEN, RECK* and *Bcl-2*. Our results were consistent with previous reports [50], [52], [55], [58] which demonstrated that up-regulation of miR-21 and decrease of *PTEN* and *RECK* as well as increase of *Bcl-2* occur in YTMLC-90 and NCI-H157 cell lines. Our studies also showed that down-regulation of miR-21 could lead to a significant increase in *PTEN* and *RECK* and a significant decrease in *Bcl-2* at both mRNA and protein level. Furthermore, our findings revealed down-regulation of miR-21 suppressed the proliferation of NSCLC cells (YTMLC-90 and NCI-H157), suggesting that miR-21 as direct regulator of *PTEN*, *RECK* and *Bcl-2* could be the key factor involved in cell proliferation in NSCLC. Interestingly, our data outline negative regulation of *PTEN* and *RECK*, but positive regulation of *Bcl-2* by miR-21 in cell proliferation. The exact mechanism of how it occurs needs to be further studied.

Results of our current study have also highlighted the importance of up-regulation of miR-21 for NSCLC cells (YTMLC-90 and NCI-H157) to develop and/or sustain the invasive phenotype. The decrease in miR-21 expression level leads to the enhanced induction of *PTEN* and *RECK*, but the reduced expression of *Bcl-2* which could further promote cell migration and invasion in NCSLC cells. Our flow cytometry data demonstrated that miR-21 silencing could induce apoptosis which was positively correlated with Bcl-2, but negatively correlated with *PTEN* and *RECK* in NSCLC cells. Furthermore, our results indicated an association between the induction of the cell cycle arrest at G_2_/M phase and *PTEN* gene expression regulated by miR-21 silencing. These results were in agreement with previous reports that *PTEN* is a regulator of the cell cycle [59]. Taken together, we have not observed any change in the levels of miR-21 and its target genes both in YTMLC-90 and NCI-H157 cells suggesting that similar regulatory mechanisms of between miR-21 and its target genes (*PTEN*, *RECK* and *Bcl-2)* involved in cell proliferation, viability, invasion, migration and apoptosis occur in GSQCLC as well as NSCLC.

**Figure 9:**
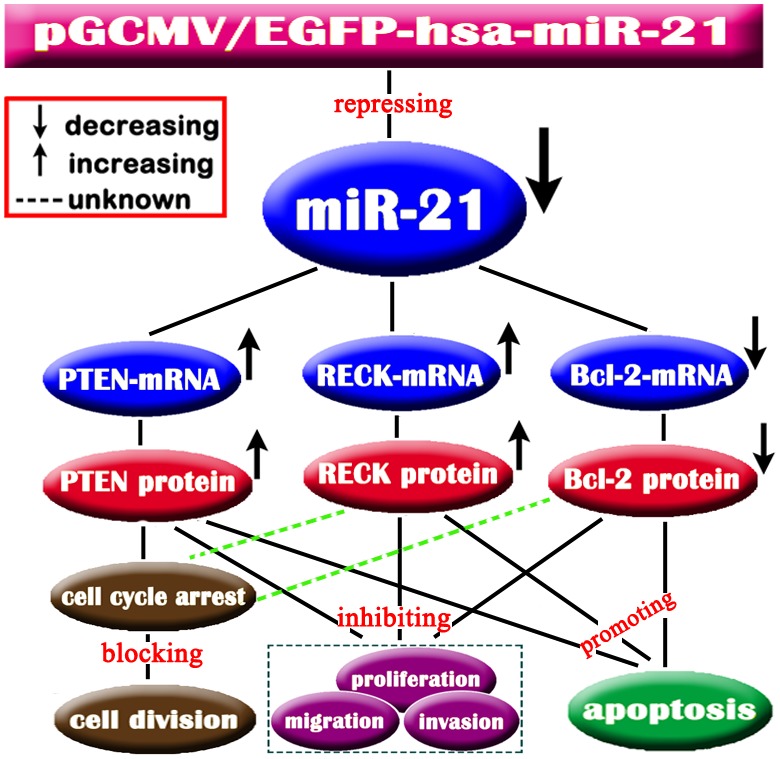
The regulatory mechanisms between miR-21 and *PTEN/RECK/Bcl-2* in non-small lung cell carcinoma cells transfected with pGCMV-hsa-miR-21. At the post-transcriptional level, *PTEN/RECK/Bcl-2* expression level was regulated by miR-21. Knockdown of miR-21 activated *PTEN* and *RECK*, and repressed *Bcl-2* at the protein level. *PTEN*, *RECK* and *Bcl-2* may have single or combined effect on NSCLC cell proliferation, migration, invasion, and apoptosis at the protein level.

In summary, the results of this study further showed the regulatory mechanism of miR-21 targeting *PTEN*, *RECK* and *Bcl-2* in NSCLC. In particular, our findings revealed that miR-21 promoted the progression of GSQCLC through negative regulation of *PTEN* and *RECK*, but positive regulation of *Bcl-2*. We propose that the molecular mechanisms that miR-21 promotes cell proliferation, viability, invasion, and apoptosis via its target genes (*PTEN*, *RECK* and *Bcl-2)* are common both in GSQCLC and other NSCLC. The possible regulatory mechanism of miR-21 can be showed in Fig. 9 [28], [29], [30], [31], [49], [50]. Furthermore, our data further underline the pivotal roles played by miR-21, provide new insight into the progression of squamous cell lung carcinoma, and also suggest that miR-21 may have potential diagnostic and therapeutic value for squamous cell lung carcinoma around the world. And what’s more, our results indicate that the tumorgenesis and progression of GSQCLC is partly similar to that of other NSCLC with respect to molecular genetics which raises doubts about the current notion of this regional-specific disease. It is hard to say whether the high incidence of squamous cell lung carcinoma in South China could only be attributed to scale-specific effects of environmental variables in the area or specific molecular genetic variation. We need to further study the molecular mechanisms of GSQCLC tumorgenesis and progression.

## Supporting Information

File S1 **Figure S1, Construction and identification of pGCMV/EGFP-hsa-miR-21 interference plasmid.** **A.** Plasmid profile. **B.** The electrophoretic identification result of plasmid after double digestion showed that the objective gene sequence (120 bp) was contained in pGCMV/EGFP-hsa-miR-21 interference plasmid. “M” is maker, “1” means double digestion, and “2” means no digestion. **C.** The sequencing chromatogram of plasmid before transformation. **D.** The sequencing chromatogram of plasmid after transformation. Objective gene sequences were marked in red lines. **Figure S2, The expression of miR-21 in NSCLC cells transfected for 24 h, 48 h and 72 h.** The expression level of miR-21 was analyzed by quantitative real-time PCR. The best transfection time was at 48 h, which had a higher inhibitory effect than 24 h and 72 h for both YTMLC-90 and NCI-H157 cell lines. n = 3, *p<0.05, **p<0.01 versus corresponding control, NC means negative control (Cells transfected with pGCMV/EGFP-hsa-miR-NC plasmid), RQ means relative quantitation. **Figure S3, miR-21 regulated protein level of its targets in NSCLC cells.** Successful cancer cell transfection after 48 h was determined using western blot assay. The *β-actin* level was also measured as a reference gene and BEAS-2B was served as a control. n = 3, *p<0.05, **p<0.01 versus corresponding control or negative control (Cells transfected with pGCMV/EGFP-hsa-miR-NC plasmid). **Figure S4, Concentration gradient of** ***PTEN*** **protein in YTMLC-90 and BEAS-2B cells.** Four gradients were set up, including 2.5 µl, 5.0 µl, 7.5 µl and 10.0 µl, to detect the protein level in YTMLC-90 cells. The results showed that *PTEN* protein was not expressed in YTMLC-90 cells. BEAS-2B was served as a control. **Table S1, Supporting data for** **Fig. 1** **.** The relative quantification (RQ) was calculated through RQ = 2^−ΔΔCt^ after normalization to reference gene. # represents means of RQ and all data represent means of triplicates ± SD, n = 3, Ct is cycle threshold. **Table S2, Supporting data for** **Fig. 4** **-A.** Values of each group were shown to support line charts in Fig. 4-A (Table S2 and Fig. 4-A have the same data). # represents means of optical density values and all data represent means ± SD. n = 6, *p<0.05, **p<0.01, ***p<0.001 versus corresponding control. u-t means un- transfected, p-NC means pGCMV-hsa-miR-NC plasmid. p-21 means pGCMV-hsa-miR-21. **Table S3, Supporting data for** **Fig. 6** **.** Values of each group were shown to support histograms in Fig. 6 (Table S3 and Fig. 6 have the same data) and the average number of cell invasion was calculated from 5 random views. # represents means of cell number and all data represent means of quintuplicates ± SD.
(DOC)
