# Supplementary material for: MicroRNA-21 (miR-21) Regulates Cellular Proliferation, Invasion, Migration, and Apoptosis by Targeting PTEN, RECK and Bcl-2 in Lung Squamous Carcinoma, Gejiu City, China
Source: PLoS One. 2014 Aug 1;9(8):e103698. doi: 10.1371/journal.pone.0103698 (PMC4118890; doi:10.1371/journal.pone.0103698)
Supplement: File S1 — Figure S1, Construction and identification of pGCMV/EGFP-hsa-miR-21 interference plasmid. A. Plasmid profile. B. The electrophoretic identification result of plasmid after double digestion showed that the objective gene sequence (120 bp) was contained in pGCMV/EGFP-hsa-miR-21 interference plasmid. “M” is maker, “1” means double digestion, and “2” means no digestion. C. The sequencing chromatogram of plasmid before transformation. D. The sequencing chromatogram of plasmid after transformation. Objective gene sequences were marked in red lines. Figure S2, The expression of miR-21 in NSCLC cells transfected for 24 h, 48 h and 72 h. The expression level of miR-21 was analyzed by quantitative real-time PCR. The best transfection time was at 48 h, which had a higher inhibitory effect than 24 h and 72 h for both YTMLC-90 and NCI-H157 cell lines. n = 3, *p<0.05, **p<0.01 versus corresponding control, NC means negative control (Cells transfected with pGCMV/EGFP-hsa-miR-NC plasmid), RQ means relative quantitation. Figure S3, miR-21 regulated protein level of its targets in NSCLC cells. Successful cancer cell transfection after 48 h was determined using western blot assay. The β-actin level was also measured as a reference gene and BEAS-2B was served as a control. n = 3, *p<0.05, **p<0.01 versus corresponding control or negative control (Cells transfected with pGCMV/EGFP-hsa-miR-NC plasmid). Figure S4, Concentration gradient of PTEN protein in YTMLC-90 and BEAS-2B cells. Four gradients were set up, including 2.5 µl, 5.0 µl, 7.5 µl and 10.0 µl, to detect the protein level in YTMLC-90 cells. The results showed that PTEN protein was not expressed in YTMLC-90 cells. BEAS-2B was served as a control. Table S1, Supporting data for Fig. 1 . The relative quantification (RQ) was calculated through RQ = 2−ΔΔCt after normalization to reference gene. # represents means of RQ and all data represent means of triplicates ± SD, n = 3, Ct is cycle threshold. Table S2, Supporting data for Fig. 4 [file pone.0103698.s001.doc]

**File S1：Supporting Information**

**Fig. S1**


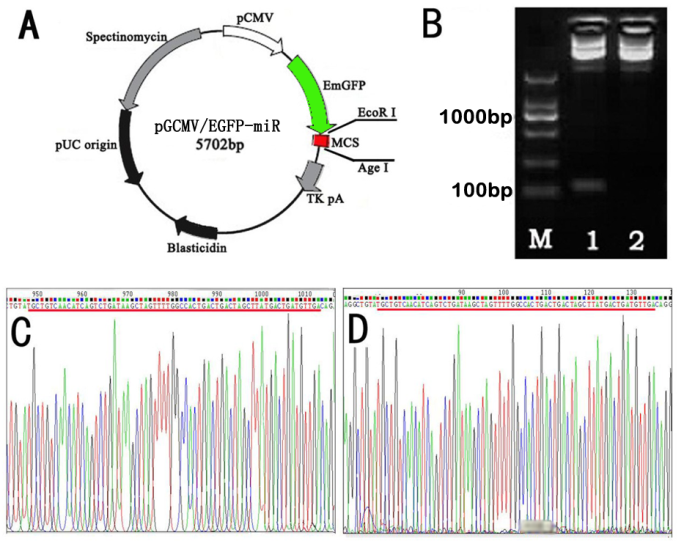


**Construction and identification of pGCMV/EGFP-hsa-miR-21 interference plasmid.** **A.** Plasmid profile. **B.** The electrophoretic identification result of plasmid after double digestion showed that the objective gene sequence (120bp) was contained in pGCMV/EGFP-hsa-miR-21 interference plasmid. “M” is maker, “1” means double digestion, and “2” means no digestion. **C.** The sequencing chromatogram of plasmid before transformation. **D.** The sequencing chromatogram of plasmid after transformation. Objective gene sequences were marked in red lines.

**Fig. S2**


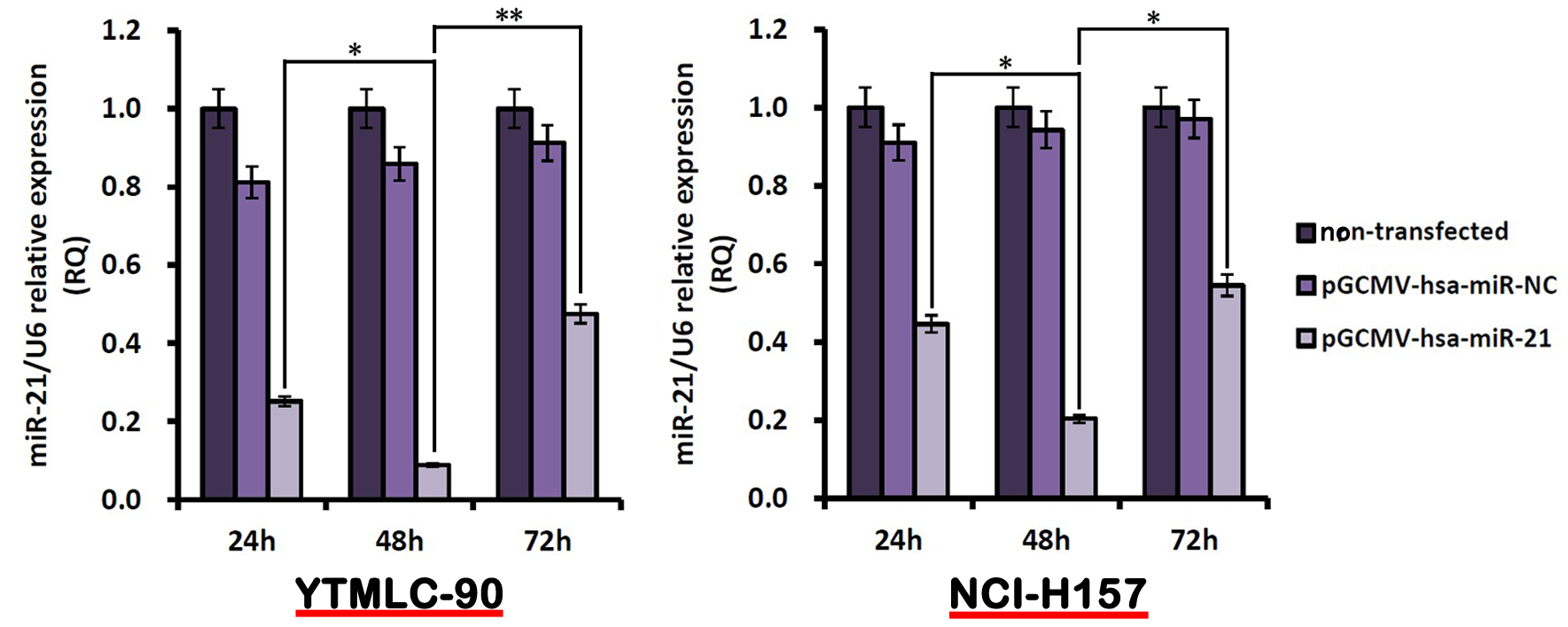


**The expression of miR-21 in NSCLC cells transfected for 24h, 48h and 72h.** The expression level of miR-21 was analyzed by quantitative real-time PCR. The best transfection time was at 48h which had a higher inhibitory effect than 24h and 72h for both YTMLC-90 and NCI-H157 cells. n = 3, *p <0.05, **p < 0.01 versus corresponding control, NC means negative control (transfected with pGCMV/EGFP-hsa-miR-NC plasmid), RQ means relative quantitation.

**Fig. S3**


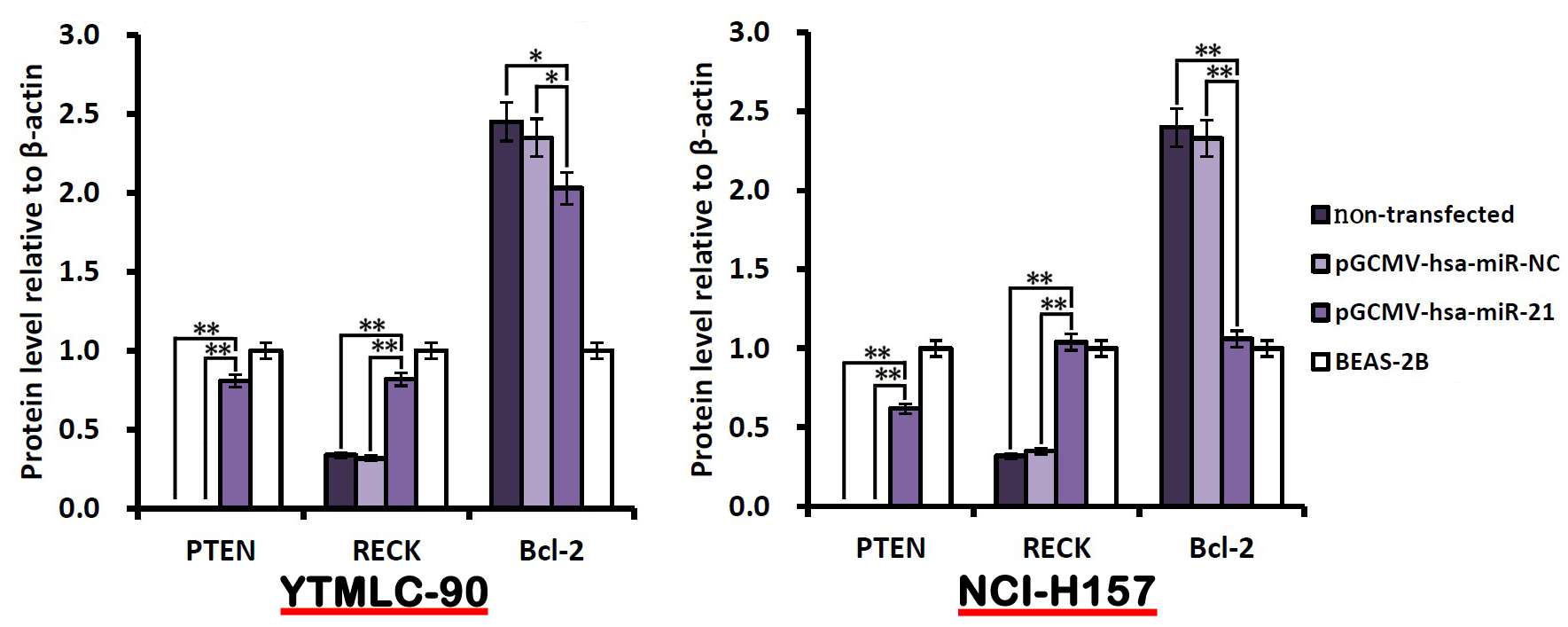


**miR-21 regulated protein level of its targets in NSCLC cells.** Cancer cells transfected for 48h was determined using western blot assay. The β-actin level was also measured as a reference gene and BEAS-2B was served as a control. n = 3, *p < 0.05, **p < 0.01 versus corresponding control, NC means negative control (transfected with pGCMV/EGFP-hsa-miR-NC plasmid).

**Fig. S4**


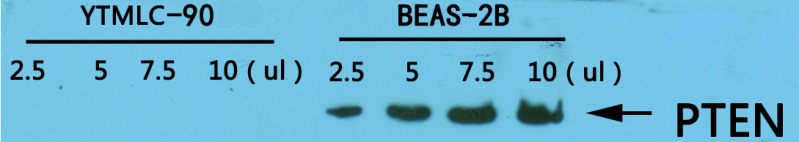


**Concentration gradient of PTEN protein in YTMLC-90 and BEAS-2B cells.** Four gradients were set up, including 2.5ul, 5.0ul, 7.5ul and 10.0ul, to detect the protein level in YTMLC-90 cells. The result showed that PTEN protein was not expressed in YTMLC-90 cells. BEAS-2B was served as a control.

**Table S1**

| **Cell line** | **ΔCt** | **ΔΔCt** | **RQ^#^** | **Log_10_(RQ^#^)** |
| --- | --- | --- | --- | --- |
| **A549** | -6.818 ± 1.833 | -3.426 ± 1.784 | 10.748 ± 0.290 | 1.0313 ± 0.0134 |
| **GLC** | -5.181 ± 0.123 | -1.789 ± 0.074 | 3.456 ± 0.950 | 0.5386 ± 0.0223 |
| **NCI-H157** | -6.05 ± 0.032 | -2.658 ± 0.017 | 6.312 ± 0.988 | 0.8002 ± 0.0053 |
| **H460SM** | -4.195 ± 0.168 | -0.803 ± 0.119 | 1.745 ± 0.921 | 0.2418 ± 0.0357 |
| **YTMLC-90** | -6.442 ± 0.259 | -3.05 ± 0.210 | 8.282 ± 0.865 | 0.7228 ± 0.0630 |
| **BEAS-2B** | -3.392 ± 0.049 | 0 | 1 | 0 |
| **NCI-H460** | -4.606 ± 0.059 | -1.214 ± 0.010 | 2.320± 0.993 | 0.3655 ± 0.0031 |
| **XWLC-05** | -4.756 ± 0.614 | -1.364 ± 0.565 | 2.574 ± 0.676 | 0.4106 ± 0.1701 |

**Supporting data for Fig. 1**. The relative quantification (RQ) were calculated through RQ = 2^-ΔΔCt^ after normalization to reference gene. # represents means of RQ and all data represent means of triplicates ± SD, n = 3, Ct is cycle threshold.

**Table S2**

|  | **Optical density (OD^#^ )value** | | | | | |
| --- | --- | --- | --- | --- | --- | --- |
|  | **YTMLC-90** | | | **NCI-H157** | | |
| **Day** | n-t | p-NC | p-21 | n-t | p-NC | p-21 |
| 1 | 0.304 ± 0.008 | 0.299 ± 0.033 | 0.244 ±0.010^**^ | 0.135 ± 0.013 | 0.139 ± 0.006 | 0.135 ± 0.025 |
| 2 | 0.469 ± 0.037 | 0.413 ± 0.017 | 0.330 ± 0.016^**^ | 0.217 ± 0.017 | 0.221 ± 0.013 | 0.172 ± 0.009^*^ |
| 3 | 0.690 ± 0.033 | 0.649 ± 0.044 | 0.468 ± 0.032^***^ | 0.508 ± 0.030 | 0.481 ± 0.027 | 0.208 ± 0.008^***^ |
| 4 | 1.019 ± 0.048 | 0.998 ± 0.084 | 0.575 ± 0.044^***^ | 0.949 ± 0.077 | 0.897 ± 0.039 | 0.318 ± 0.014^***^ |
| 5 | 1.490 ± 0.055 | 1.318 ± 0.088 | 0.845 ± 0.101^***^ | 1.540 ± 0.040 | 1.488 ± 0.094 | 0.658 ± 0.023^***^ |
| 6 | 1.899 ± 0.018 | 1.686 ± 0.466 | 1.023 ± 0.195^***^ | 2.231 ± 0.292 | 2.246 ± 0.090 | 1.318 ± 0.054^***^ |
| 7 | 2.297 ± 0.029 | 2.225 ± 0.094 | 1.429 ± 0.063^***^ | 2.745 ± 0.095 | 2.770 ± 0.040 | 1.756 ± 0.050^***^ |

**Supporting data for Fig. 4-A.** Values of each group were showed to support line charts in Fig. 4-A (Table S2 and Fig. 4-A have the same data). # represents means of optical density values and all data represent means ± SD. n = 6, *p < 0.05, **p < 0.01, ***p < 0.001 versus corresponding control, n-t means non- transfected, p-NC means pGCMV-hsa-miR-NC, p-21 means pGCMV-hsa-miR-21.

**Table S3**

| **Cell line** | **Group** | **n** | **Cell number^#^** | **p value** | ***F* value** |
| --- | --- | --- | --- | --- | --- |
| **YTMLC-90** | non-transfected | 5 | 84.0 ± 6.44 | —— | *F*_(2,12)_ = 86.706 |
|  | pGCMV-hsa-miR-NC | 5 | 81.0 ± 5.79 | 0.422 |  |
|  | pGCMV-hsa-miR-21 | 5 | 21.4 ± 4.77 | 0.000 |  |
| **NCI-H157** | non-transfected | 5 | 84.6 ± 3.36 | —— | *F*_(2,12)_ = 108.561 |
|  | pGCMV-hsa-miR-NC | 5 | 83.2 ± 3.11 | 0.679 |  |
|  | pGCMV-hsa-miR-21 | 5 | 41.8 ± 7.79 | 0.000 |  |

**Supporting data for Fig. 6**. Values of each group were showed to support histograms in Fig. 6 (Table S3 and Fig. 6 have the same data) and the average number of invasion cells was calculated from 5 random views. # represents means of cell number and all data represent means of quintuplicates ± SD.
